# Genetics of Thyroid Function: Relevance for Biology and Disease Management

**DOI:** 10.1210/clinem/dgaf491

**Published:** 2025-11-18

**Authors:** Rosalie B T M Sterenborg, Robin P Peeters, Edward Visser, Aleksander Kuś, Jan W A Smit, Alexander Teumer, Marco Medici

**Affiliations:** Department of Internal Medicine, Division of Endocrinology, Radboud University Medical Center, 6525 GA Nijmegen, the Netherlands; Department of Internal Medicine, Academic Center for Thyroid Diseases, Erasmus Medical Center, 3015 GD Rotterdam, the Netherlands; Department of Internal Medicine, Academic Center for Thyroid Diseases, Erasmus Medical Center, 3015 GD Rotterdam, the Netherlands; Department of Internal Medicine, Academic Center for Thyroid Diseases, Erasmus Medical Center, 3015 GD Rotterdam, the Netherlands; Department of Internal Medicine and Endocrinology, Medical University of Warsaw, 02-507 Warsaw, Poland; Department of Internal Medicine, Division of Endocrinology, Radboud University Medical Center, 6525 GA Nijmegen, the Netherlands; DZHK (German Center for Cardiovascular Research), Partner Site Greifswald, 17475 Greifswald, Germany; Department of Population Medicine and Lifestyle Diseases Prevention, Medical University of Bialystok, 15-269 Bialystok, Poland; Department of Psychiatry and Psychotherapy, University Medicine Greifswald, 17489 Greifswald, Germany; Department of Internal Medicine, Academic Center for Thyroid Diseases, Erasmus Medical Center, 3015 GD Rotterdam, the Netherlands

**Keywords:** thyroid, genetics, genome-wide association study, mendelian randomization, polygenic score, prediction

## Abstract

**Context:**

Genetic factors are a major contributor to variation in thyroid function. Recent studies have partly identified the responsible common genetic variants and studied their application in unraveling thyroid (patho)physiology as well as their potential clinical use.

**Evidence Acquisition:**

This review summarizes the current state of knowledge regarding the genetic architecture of thyroid function as well as its applications to improve (patho)physiological understanding and clinical management of thyroid (dys)function.

**Evidence Synthesis:**

Genome-wide association studies (GWAS) have been successful in detecting numerous genetic variants affecting variation in thyrotropin (TSH), free thyroxine, and triiodothyronine concentrations. Subsequent emerging high-throughput in silico and in vitro strategies are of particular value in unraveling functionality of these novel genes and its genetic variants. Translational methods such as mendelian randomization (MR) and polygenic scores (PGSs) can provide important insights into causal associations or susceptibility to disease. Moreover, PGSs show potential in adjusting personalized TSH reference ranges by distinguishing between individual hypothalamic-pituitary-thyroid–axis set-point effects and (subclinical) thyroid dysfunction.

**Conclusion:**

Functional characterization of the associated genes and variants in GWAS is warranted as the majority are located in genes with a yet unknown role in thyroid hormone physiology. Integration of multi-omics data and optimalization of translational applications such as MR and PGS show potential to further unravel the underlying molecular mechanisms and pave the way for incorporation of genetics in personalized management of thyroid diseases.

Adequate thyroid function plays a key role in the maintenance of whole-body metabolism (1). Serum thyrotropin (TSH) and free thyroxine (FT4) concentrations are routinely used to assess thyroid function of an individual in everyday clinical practice. Subclinical thyroid dysfunction is biochemically characterized by TSH levels outside the population-based reference range with FT4 levels within the reference range. It has been demonstrated that the term “subclinical” may be not justified, as, similar to overt thyroid disease, subclinical thyroid dysfunction is also associated with potentially serious clinical outcomes ranging from cardiometabolic disorders (ie, dyslipidemia, atrial fibrillation, stroke, and cardiovascular morbidity), mood disorders, to even mortality (2-6). Moreover, it has been shown that minor differences in thyroid function within the population-based reference range are also associated with adverse outcomes, including atrial fibrillation, stroke, as well as type 2 diabetes, depression, and dementia (7). This is of particular interest as diagnosis and treatment of thyroid dysfunction are currently based on wide population-based reference ranges.

A seminal paper by Andersen et al (8) showed that the interindividual variation of circulating TSH, FT4, and triiodothyronine (T3) levels in euthyroid individuals is substantially larger than the intraindividual variation, suggesting that every individual has a unique hypothalamic-pituitary-thyroid (HPT)-axis set-point. Subsequent heritability studies showed that the majority (57%-71%) of the variation in TSH, FT4, and T3 concentrations is genetically determined (9). Thyroid function is considered a polygenic trait, that is, it is determined by multiple common variants (polymorphisms) with small effect sizes as well as rare variants with large effect sizes (mutations). This review provides a systematic overview of the recent discoveries of polymorphisms associated with variation in thyroid function, with a particular focus on thyroid function within the reference range, as well as their use to understand thyroid (patho)physiology and their potential application in personalizing the clinical management of thyroid dysfunction.

## Methods

While this is not a systematic review, we performed an extensive PubMed search between January 1, 1996, and December 1, 2024, using the following search strategy: (“thyroid gland”[MeSH Terms] OR (“thyroid”[All Fields] AND “gland”[All Fields]) OR “thyroid gland”[All Fields] OR “thyroid”[All Fields] OR “thyroid usp”[MeSH Terms] OR (“thyroid”[All Fields] AND “usp”[All Fields]) OR “thyroid usp”[All Fields] OR “thyroids”[All Fields] OR “thyroid”[All Fields] OR “thyroidal”[All Fields] OR “thyroideal”[All Fields] OR “thyroidism”[All Fields] OR “thyroiditis”[MeSH Terms] OR “thyroiditis”[All Fields] OR “thyroiditides”[All Fields]) AND (“genome wide association study”[MeSH Terms] OR (“genome wide”[All Fields] AND “association”[All Fields] AND “study”[All Fields]) OR “genome wide association study”[All Fields] OR “gwas”[All Fields]) AND (“genetic”[All Fields] OR “genetical”[All Fields] OR “genetically”[All Fields] OR “genetics”[MeSH Subheading] OR “genetics”[All Fields] OR “genetics”[MeSH Terms]), yielding 622 papers. For the different subsections of this review, relevant papers were extracted and findings summarized.

## Genetic Architecture of Thyroid Function

In the last 3 decades, many studies have investigated the influence of genetic variants on thyroid function (7, 10-16). Particularly, small variants in DNA, also called single-nucleotide variants (SNVs) or single-nucleotide polymorphisms (SNPs; in case of an allele frequency >1%) have been examined in population-based or patient cohorts.

Initially many so-called candidate gene studies were performed, investigating genetic variants in a limited number of genes with a known role in thyroid hormone (TH) regulation, such as *TSHR* encoding the TSH receptor, and *DIO1* and *DIO2* encoding TH deiodinases (14, 17-20). These studies were succeeded by a more comprehensive candidate gene study, examining genetic variation in 68 genes involved in TH regulation (21). This study yielded limited new findings, namely replicating known associations in *PDE8B*, *THRB*, and *DIO1* and identifying only one novel association with FT4 near the *FOXE1* gene.

Thanks to the introduction of high-throughput genotyping techniques that can rapidly determine many SNPs across the entire genome in many samples, the field turned toward performing genome-wide association studies (GWAS). In GWAS, hundreds of thousands and nowadays even millions of genetic variants are tested across the entire genome of large samples of individuals to identify genotype-phenotype associations. While the first TSH GWAS identified only 1 variant in the *PDE8B* gene, later GWAS including larger sample sizes were able to identify up to 99 genome-wide significant independent associations with TSH, 31 associations with FT4, and 1 with FT3 levels (7, 11, 12, 15, 16, 22). Although the majority of identified genetic variants are located near or within genes with a yet unknown role in TH regulation, genes with a known role in TH regulation likely nest multiple independent variants affecting TSH and TH concentrations. Therefore, a recent large-scale candidate gene study investigated the effects of common genetic variation in 96 genes with a known role in TH regulation on serum TSH and FT4 concentrations within the reference range using GWAS summary statistics (10). Despite the identification of numerous novel independent genetic associations in these genes, these variants still explained only 1.9% (SD = 0.4, TSH) to 2.6% (SD = 0.4, FT4) of the variation in thyroid function within the reference range, suggesting that many yet unknown pathways in TH regulation remain to be discovered.

**Figure 1.**
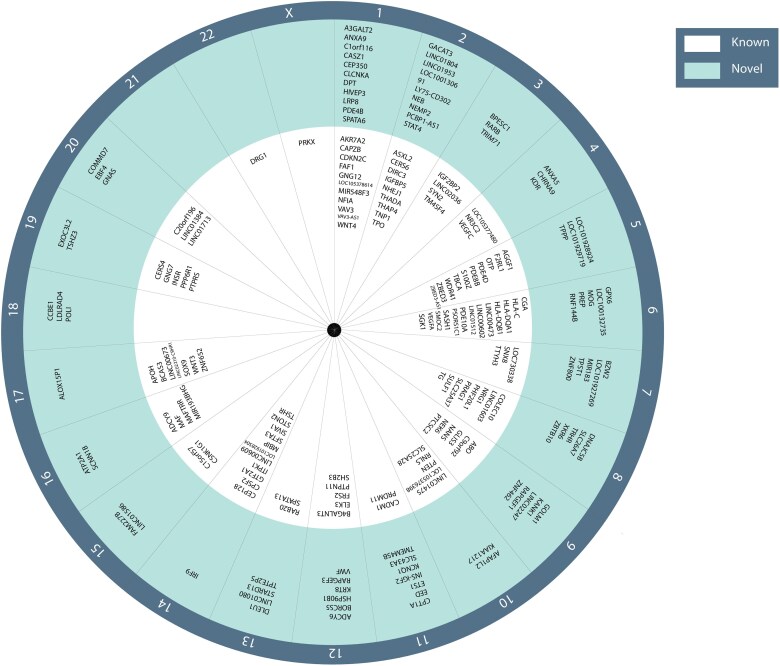
Overview of genome-wide significant reference range thyrotropin-associated variants identified in genome-wide association studies (GWAS). The nearest gene around the lead variant is assigned as the locus name. A locus was defined as known when correlated (*R^2^* > 0.1) with a previously known variant within a ±10-Mb distance (11, 12). Loci per chromosome are displayed in the inner band if previously discovered (11, 12) and in the outer band when discovered in the most recent GWAS (23).

**Figure 2.**
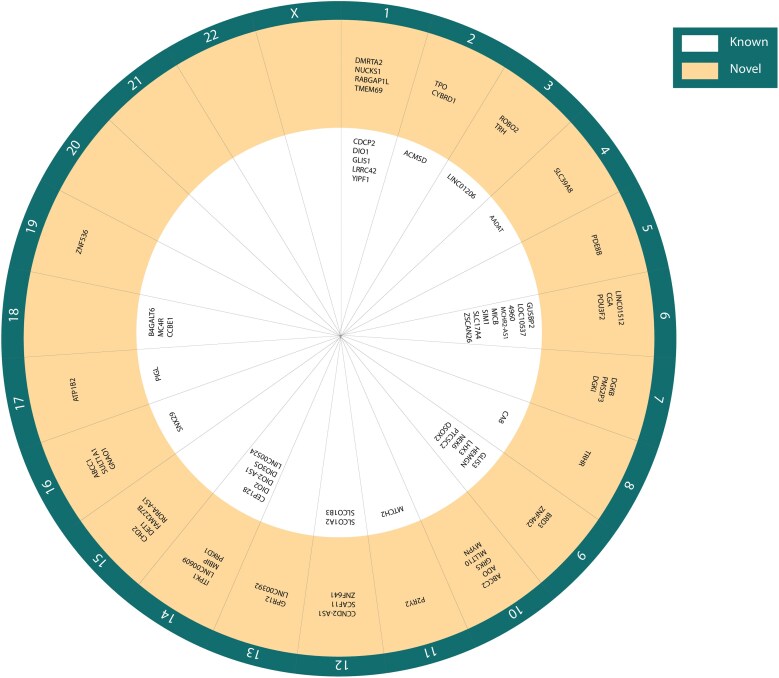
Overview of genome-wide significant free thyroxine–associated variants identified in genome-wide association studies (GWAS). The nearest gene around the lead variant is assigned as the locus name. A locus was defined as known when correlated (*R^2^* > 0.1) with a previously known variant within a ±10-Mb distance (11, 12). Loci per chromosome are displayed in the inner band (white) if previously discovered (11, 12) and in the outer band (blue) when discovered in the most recent GWAS (23).

Most recently, a large GWAS testing the effects of 7 888 577 genetic variants on reference range thyroid function in 271 040 individuals of European ancestry identified 406 genetic variants for different markers of thyroid function (23). The genome-wide significant variants (*P* < 5E-08) accounted for 14.1% (TSH), 6.0% (FT4), 1.1% (FT3), 9.5% (total T3 [TT3]), 2.7% (FT3/FT4 ratio), and 7.0% (TT3/FT4 ratio) of the total variation in serum concentrations. Compared to the previous thyroid function GWAS, the almost doubling to tripling of sample sizes for TSH and FT4 respectively led to a substantial increase in explained variance of 2.2% (FT4) and 5.6% (TSH) (11). An overview of the identified variants of the current largest GWAS for TSH and FT4 compared to previous studies is provided in Figs. 1 and 2 (11, 12).

Non-European ancestries are currently underrepresented in genetic analyses. However, a GWAS of thyroid function, dysfunction, and autoimmunity has recently been performed in 85 421 Chinese pregnant women (24). For normal range thyroid function, applying pregnancy-specific reference ranges, 22 of the 37 detected loci for TSH and 9 of the 22 detected loci for FT4 overlapped with the currently largest nonpregnant thyroid function GWAS (23). This substantial discrepancy is likely explained for an important part by differences in ethnic background (Asian vs predominantly European) and the effect of pregnancy on TH regulation.

Importantly, heritability estimates vary with phenotype precision but also with other factors such as sex and age. Different approaches exist to calculate heritability estimates (eg, linkage disequilibrium score regression using summary statistics, genome-wide complex trait analysis using individual-level data, or a simplified equation including allele frequencies, effect size and SE estimates), with all strategies considering additive effects of variants for calculating SNP-based heritability (h^2^). The genetic contribution of all common genetic variants to the total variation in thyroid function using the linkage disequilibrium score is estimated at 30.7% ($h_{\mathrm{SNPs}}^{2}$) and 23.3% for TSH and FT4 concentrations, respectively, leading to a current missing heritability of 16.6% points for TSH and 17.3 for FT4 that could be explained by common variants (10).

**Figure 3.**
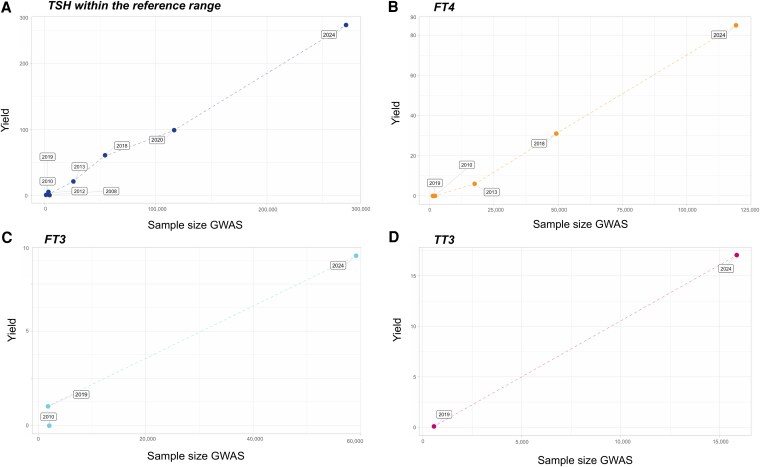
Overview of reference range thyrotropin (TSH, panel A), free thyroxine (FT4, panel B), free triiodothyronine (FT3, panel C), and total triiodothyronine (TT3, panel D) genome-wide association studies (GWAS) approaches and the respective sample sizes and yield. Required sample sizes (N, x-axis) for identifying the associated number of genome-wide significant variants (yield, y-axis) are displayed for the different thyroid function-related traits: TSH (n > 5,000, reference range TSH GWAS) (11, 12, 14, 16, 23, 25, 26), FT4 (11, 14, 16, 23), FT3 (14, 16, 23), and TT3 (15, 23).

**Figure 4.**
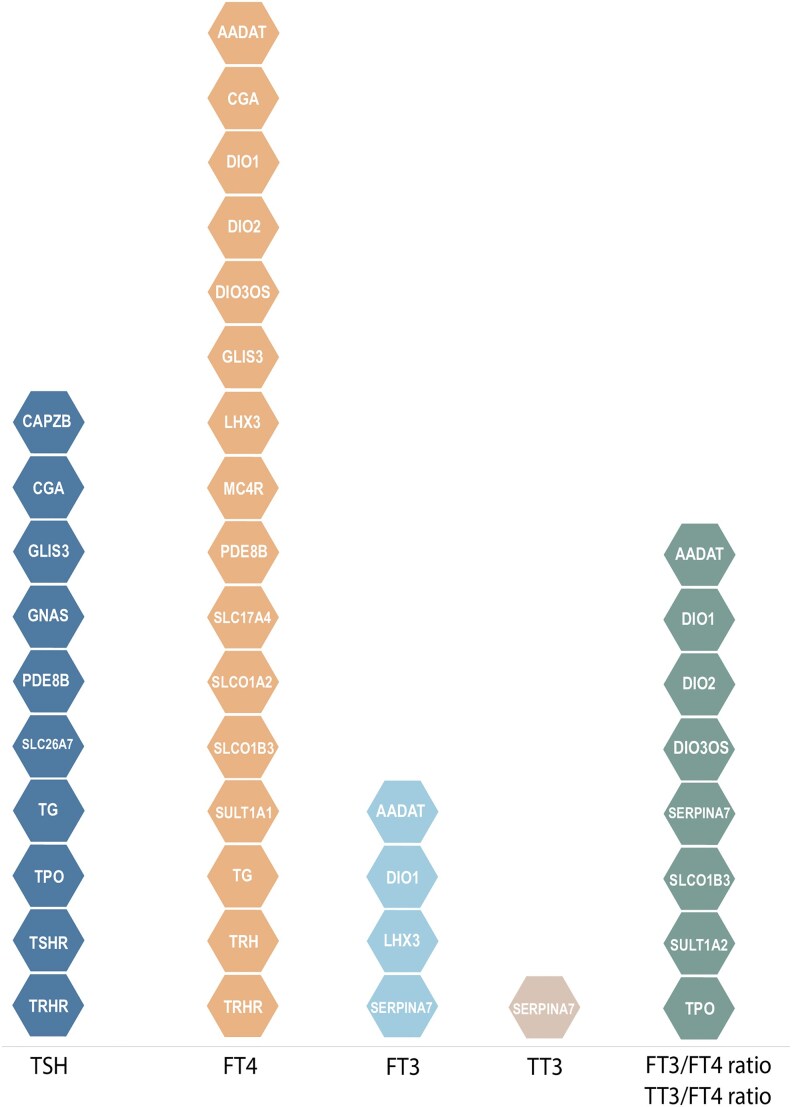
Genome-wide association studies discovered loci in/near known thyroid hormone–regulating candidate genes. Selection of genome-wide significant variants affecting thyroid function within the reference range (23) that are assigned to a gene with a known role in thyroid hormone regulation (10).

In conclusion, GWAS sample sizes need to be increased to further narrow the remaining gap in missing heritability explained by common variants (Fig. 3). When extrapolating the trends indicated in Fig. 3, at least a tripling of the sample size is necessary for TSH (±1 million individuals), as the yet unidentified variants will include common variants with smaller effect sizes or less common variants with larger effect sizes, which will therefore individually not contribute much to the explained variance. Next, there is a large difference between the estimates of the genetic heritability explained by common variants and the estimated genetic heritability explained by all (common + rare) genetic variants (31% vs 71%, respectively). To date, the influence of rare variation has been sparsely investigated in the thyroid field (13), which can be studied in GWAS with substantially larger sample size or in exome or whole-genome sequencing studies. Indeed, such an approach has been shown to be successful in identifying rare variants for other outcomes such as body mass index (BMI) (27, 28), height (29), and blood pressure (30). Also gene-gene or gene-environment interactions may contribute to the missing heritability gap (eg, as shown for BMI (31)), which would also require large sample sizes using biobanks or consortia, such as the ThyroidOmics Consortium, which currently includes more than 50 cohorts that possess genetic and phenotypic (thyroid function) data (32). As most GWAS rely on only one thyroid function measurement, cohorts including repeated TH measurements would also be of interest to further decrease the phenotypic variation, thereby increasing its overall yield. Unfortunately, to date only a minority of the cohorts have repeated TH measurements available. Finally, also in the latest GWAS the minority of the discovered loci were found in or near genes with a known role in TH regulation, for example, genes important in TSH signaling (*CGA*, *GNAS*), deiodinases important for the conversion of T4 to T3 (*DIO1*, *DIO2*), TH binding protein *SERPINA7* encoding thyroxine binding globulin, central HPT-axis hormones and receptors (*TRH*, *TRHR*, *TSHR*), and TH transporters (*SLCO1B3*, *SLCO1A1*, *SLC17A4*), signifying the need for further identification of the function of the yet unknown (causal) genes or mechanisms in TH regulation in post-GWAS strategies (Fig. 4).

## Use of Genetic Markers in Unraveling (Patho)Physiology

### Functional Characterization of Identified Genes and Variants

As aforementioned, the majority of identified genetic markers are located in or near genes with an unknown role in TH regulation. Therefore, functional follow-up studies are essential to understand the underlying biological mechanism. The 2018 thyroid function GWAS (11) is a good example of such an effort: This GWAS identified several genetic variants associated with FT4 levels, and for 2 genes it was hypothesized that these could be a novel TH transporter (*SLC17A4*) and TH metabolizing enzyme (*AADAT*). Indeed, in vitro studies have demonstrated that SLC17A4 facilitates the transport both of T3 and T4 and to a lesser extent also the transport of the inactive metabolites, that is, reverse T3 and 3,3′-diiodothyronine (T2) (33), while in parallel AADAT was shown to convert T4 and T3 to their pyruvic acid metabolites.

In addition, for many of the recently identified variants in silico functionality testing has been performed. An example includes colocalization analyses that use messenger RNA expression data from the GTEx Project database to assess whether the effect of a genetic variant operates via differential gene expression on the trait of interest (23). This is of particular importance as the nearest gene of a specific SNP does not necessarily have to be the causal gene responsible for the observed association. Besides genetic variants, other important modifiers of gene expression include epigenetic mechanisms such as histone modification or DNA methylation. Many cohorts involved in previous studies also have other “omics” data available now, such as blood DNA methylation profiles, transcriptomics, and proteomics. These data can be successfully integrated with the available genomics data, as exemplified by a recent epigenome-wide association studies, showing that besides the direct influence of TH on gene transcription, TH can also affect *KLF9* transcription via DNA methylation (34, 35). Other examples include a recent proteome-wide association study and transcriptome-wide association studies revealing novel thyroid function–associated genes and prioritizing candidates in identified GWAS loci (36). For example, novel independent signals were discovered in *CAPZB*, which encodes the β-subunit of the capping protein and plays a key role in TSH-induced engulfment of colloid during thyroglobulin mobilization and subsequent proteolysis of TH in the thyroid gland (36, 37).

Furthermore, microRNAs (miRNAs) are small non–protein-coding RNA sequences that can affect gene expression at a posttranscriptional level (38). Previous studies have shown that miRNAs can target deiodinases and TH receptors (39). Future studies are needed to evaluate whether other miRNAs could affect (other) key players in TH regulation, for example, by miRNA enrichment analysis using GWAS data.

The aforementioned examples illustrate that both in vitro and in silico approaches are key to further characterize identified genes and variants.

### Mendelian Randomization

Association analyses in observational studies are prone to confounding, sampling error, and reverse causation, which can undermine a robust causal inference between the exposure and outcome. An alternative and currently popular approach to test for causality is via mendelian randomization (MR) analyses. In MR, causality is tested by investigating the associations between genetically predicted exposure (in which SNPs are used as genetic instruments) and the outcome of interest (ie, disease) (40-42). The principles of this statistical approach are based on Mendel's laws of independent assortment stating that alleles are passed randomly from parents to offspring, resulting in genetic associations being less susceptible for confounding and comparable to randomization in clinical trials. However, MR has several limitations, such as pleiotropy of SNPs potentially violating the method's assumptions and the requirement of large sample sizes. Over the last few years, public availability of GWAS data has led to a wave of published MR studies, with currently considerable concerns about the quality of some of these published MR studies (43). When used and interpreted correctly, MR has proven itself as an elegant approach to provide support to the presence of a causal association between the exposure and outcome (44). For detailed MR analysis methods and sensitivity analyses, we refer to other excellent reviews (41, 42, 45).

For reference range thyroid function, several MR studies have proven causal associations with different clinical outcomes (46-63). For example, variation in genetically predicted TSH concentrations has been associated with lipid levels, atrial fibrillation, and stroke, while variation in FT4 has been associated with waist-hip ratio, type 2 diabetes mellitus, ischemic heart disease, and bipolar disorder. Besides these cardiovascular risk factors and diseases, no causal associations have yet been discovered with psychiatric disorders, such as major depressive disorder and its subtypes, schizophrenia, and borderline personality disorder (64-67).

Recently, causal associations between variation in thyroid function and clinical outcomes were verified using MR analyses in the latest GWAS project using the currently largest set of genetic variants for TSH (maximum number of 259 variants) and FT4 (maximum number 85 variants) (23). Significant associations were detected for heart rate, diastolic blood pressure, pulse pressure, cholesterol (including low-density lipoprotein and high-density lipoprotein), atrial fibrillation, and Alzheimer disease for TSH, while for FT4 associations with height, atrial fibrillation, coronary artery disease, and bipolar disorder were confirmed. The fact that part of the previously detected associations was not confirmed could be explained either by false-positive findings in previous studies or pleiotropy of the currently used large set of variants that may dilute the association (44), which should be clarified in future studies.

MR studies are also capable of discovering mediating mechanisms underlying known epidemiological associations. This is exemplified by an MR study in which variation in genetically determined TSH levels was associated with stroke (odds ratio [OR] 0.95; 95% CI, 0.91-0.99; *P* = .008). After adjustment for atrial fibrillation in multivariable MR analyses, this association disappeared (OR 1.00; 95% CI, 0.95-1.06; *P* = .86), showing that the effect of TH on stroke is mediated via atrial fibrillation (55). A later MR study showed that the causal association between genetically determined TSH and atrial fibrillation is partly mediated by height (68). Next to the pathophysiological understanding, this might also be an informative insight from a clinical perspective as height is a nonmodifiable factor in later life, and therefore correction of thyroid function in later in life is unlikely to fully correct the effect observed in observational studies (69).

Importantly, MR studies typically assume a linear (dose-response) effect of the risk factor (ie, thyroid function) on a continuous outcome or disease (on the liability scale). However, in observational studies it has been shown that instead of a linear relationship, TSH and FT4 may show U-shaped and J-shaped relations with many outcomes, for example, with cardiovascular disease events and mortality (70). Therefore, future nonlinear MR studies are of interest in which also stratum-specific exposure-outcome associations can be calculated (71). Limitations include the fact that nonlinear MR methods typically require individual-level data, large sample sizes for adequate power, and specifically developed methods, while there is currently no general consensus on which nonlinear MR is suitable for a specific setting.

Finally, other options include (re)clustering of genetic variants, for example, based on either their biological role in TH regulation (*DIO1* and *DIO2* genes (50)) or combining the TSH- and FT3-related SNPs given their overall similar effects on outcomes and the presence of high genetic correlation (23).

Taken together, if applied correctly, MR studies can provide useful information on both causality and underlying mediating pathways.

## Potential Clinical Applications

### Individual Single-Nucleotide Variations

The type 2 deiodinase is important for the conversion of T4 to T3 and is expressed in various tissues like the pituitary, central nervous system, brown adipose tissue, muscles, heart, and bone (72). *DIO2*-rs225014 is a nonsynonymous variant that results in the Thr92Ala substitution leading to decreased conversion of T4 to T3. This SNP has been associated with several outcomes in observational studies, such as type 2 diabetes, hypertension, osteoarthritis, and bipolar disorder (73). Several small clinical follow-up studies in hypothyroid patients on levothyroxine (LT4) treatment have been conducted to investigate the effect of this SNP on patient well-being and response to LT4/LT3 combination therapy (14, 74-77). Taken together, these studies show conflicting results, which may be due either to differences in sample sizes and resulting statistical power or selection of patients who could benefit most from LT4/LT3 combination therapy (ie, patients with persisting complaints despite LT4 monotherapy). More important, the large interindividual variation in LT4/LT3 combination therapy response observed in clinical practice is unlikely to be explained by only one single genetic variant but more likely by a combination of multiple variants.

### Personalized Reference Ranges for Thyrotropin

Introduced in the 1930s, generic reference ranges of biochemical analytes are based on the 2.5th to 97.5th percentiles of distribution in the healthy population. As previously discussed, the intraindividual variation in thyroid function is smaller than the interindividual variation and even variation in thyroid function within the reference range has been associated with adverse health outcomes (2, 78-82). It is therefore likely that TSH concentrations within the population-based reference range do not necessarily exclude thyroid dysfunction, while individuals with TSH concentrations above the upper limit of the population-based reference range are likely a heterogeneous group including individuals with (mild) thyroid disease, as well as nondiseased individuals with a TSH set-point at the extremes of the distribution. Currently used population-based reference ranges are therefore not able to properly distinguish between set-point effects and (mild) thyroid disease. Furthermore, minor differences in the upper limits of the reference range for TSH concentrations are also associated with LT4 prescription behavior (83). Consequently, application of population-based reference ranges may cause overtreatment and undertreatment. Therefore, several attempts have been made to personalize reference ranges for TSH based on age or sex, but these have not (yet) been universally implemented in clinics (84-90). As the majority of the variation (57%-71%) in thyroid function is determined by genetic factors (9), obtaining a personalized genetically determined reference range would be an essential step forward in overcoming the aforementioned problems that dominate current clinical practice and therapeutical decisions for (subclinical forms of) thyroid disease.

**Figure 5.**
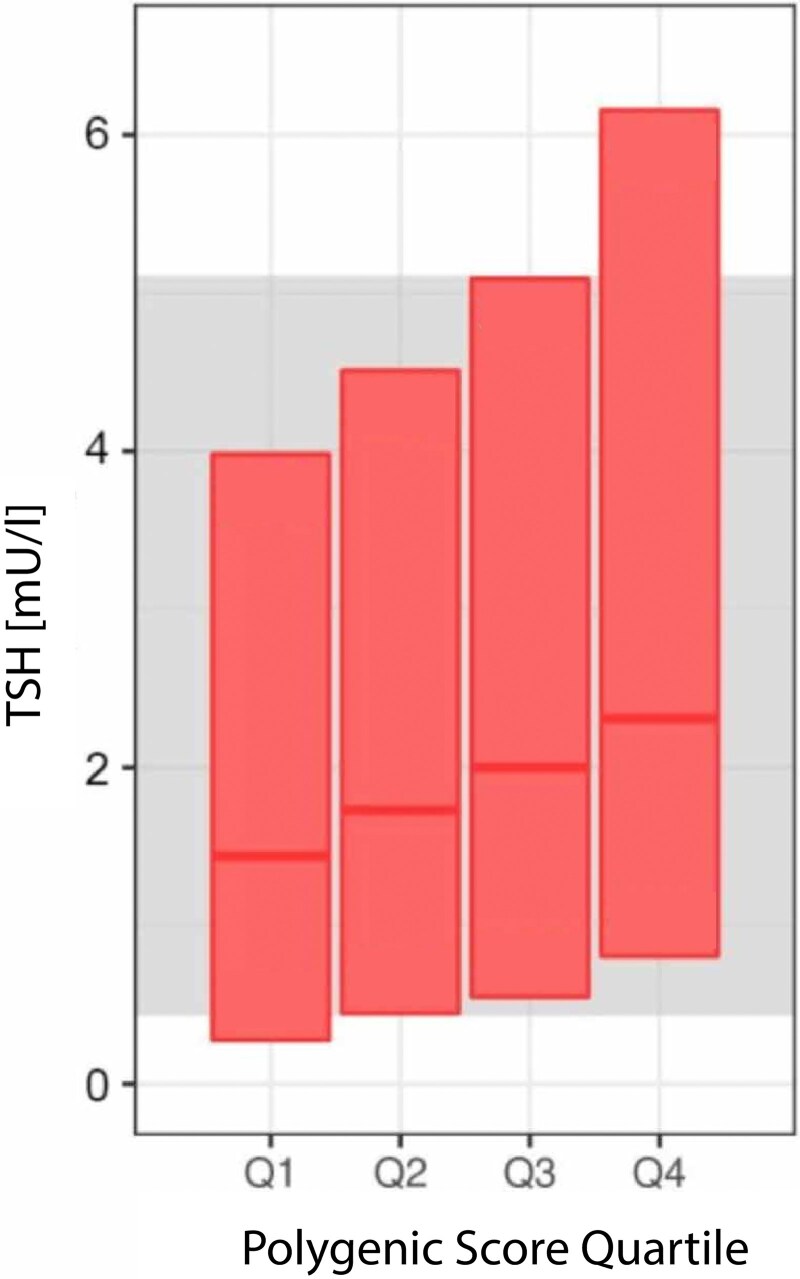
Concept of polygenic score (PGS) quartile-specific thyrotropin (TSH) reference ranges adapted from Kuś et al 2024 (91). PGS quartile-specific TSH reference ranges are shown in red, and population-based reference ranges shown in gray with data based on the Rotterdam Study (reference range, 0.43-5.11 mU/L). Solid horizontal lines correspond to median TSH concentrations in each PGS quartile.

In line with the aforementioned reasoning, a recent study tested a polygenic score (PGS) composed of common genetic variants to personalize TSH reference ranges (91). Polygenic scores are usually constructed with a predefined set of genetic variants (ie, common and/or rare variants) to calculate a score based on the weighted sum of risk alleles and the corresponding βs derived from GWAS. In most cases, only SNPs reaching genome-wide statistical significance (*P* < 5E-08) are selected (restricted-to-significant PGS). Alternatively, one may also include “all” variants of the GWAS data set regardless of being individually significant (global extended PGS), which may improve power (92, 93). Individual genetic TSH profiling was investigated in 3 different European cohorts excluding patients with known thyroid disease and thyroid peroxidase positivity. Using a PGS including 59 SNPs associated with reference range TSH concentrations (11), it was demonstrated that PGS was a much stronger predictor of individual TSH concentrations than FT4 or the other nongenetic factors age, sex, BMI, or smoking status. Substantial differences between PGS-specific TSH reference ranges were observed (eg, 2.2 mU/L difference between the upper limits of TSH concentrations in PGS quartiles 1 vs 4) (Fig. 5). The application of these personalized reference ranges reclassified up to 30.1% of the individuals to euthyroidism when compared to using conventional population-based TSH reference ranges. Moreover, individuals with a higher PGS were prescribed LT4 treatment more frequently compared to individuals with lower PGS (91). Importantly, there was no association between PGS and thyroid peroxidase antibodies concentrations below the positivity cutoff level, demonstrating that the subsequent higher TSH reference range upper limits in PGS quartiles were not driven by early stages of autoimmune hypothyroidism. This PGS included genetic variants in biologically plausible candidates, such as the *PDE8B* gene. PDE8B is important in the TSH signaling cascade affecting cyclic adenosine monophosphate levels in the thyroid (94). The genetic variants associated with TSH concentrations presumably increase PDE8B levels (23), resulting in lower cyclic adenosine monophosphate levels in response to TSH. As a response, higher TSH concentrations are required to maintain adequate TH levels (73).

As the PGS explained up to 11.1% of the variation in TSH, future studies should investigate whether this PGS can be improved by inclusion of lower frequency variants (91). Furthermore, PGS based on genetic instruments from European GWAS have reduced predictive performance in individuals of non-European ancestry (95). Multiancestry GWAS on reference range thyroid function have not yet been conducted, which should be the first step in identifying SNPs associated with reference range thyroid function in non-European ancestries.

However, although the PGS on TSH reference ranges was much more strongly associated with TSH than with FT4 levels, was not associated with thyroid peroxidase antibodies, and did not include SNPs that were significant hits in the FT4 GWAS, still the included SNPs have been associated with clinical end points (such as atrial fibrillation and lipid levels (48, 51, 55)) in MR studies. This paradox needs further discussion: Ideally, the PRS should include only pure TSH set-point SNPs with no effect on thyroid status. The mentioned results of MR studies argue against this, even though the actual effects are small. However, instead of having either pure TSH set-point or pure thyroid dysfunction effects, SNPs affecting TSH levels more likely cover a continuous scale ranging from pure set-point variants to variants with also minor effects on thyroid function. In the end, it would be reasonable to judge a TSH value above the population-based reference range differently in an individual enriched with multiple variants affecting TSH set-point than in an individual carrying no such variants. Future studies should focus on further optimalization of the prediction models including investigating its effects on clinical end points.

### Polygenic Risk Score and Disease

In the thyroid field, limited PGS studies have been performed. PGSs using effect estimates for reference range TSH-associated variants have been calculated for hypothyroidism, hyperthyroidism, and thyroid cancer (11, 23). Using a set of 61 TSH genome-wide significant variants, enriched individuals had a higher risk of developing hypothyroidism and lower risk of hyperthyroidism: Comparing the highest with the lowest quartiles, the OR for hypothyroidism and hyperthyroidism were 2.53 (*P* = 6.8 × 10^−32^) and 0.19 (*P* = 9.8 × 10^−31^), respectively (11). However, these results should be interpreted with caution as these PGSs include genetic variants that are associated with thyroid function within the reference range and therefore could represent a set-point effect rather than thyroid disease. Furthermore, a PGS including 259 TSH-associated genome-wide significant variants showed that a higher PGS quartile was associated with a lower risk of thyroid cancer (OR 0.82; *P* = 1.0 × 10^−7^). Opposite effects for FT3 were observed, with each higher PRS quartile showing a higher risk of thyroid cancer (OR = 1.16; *P* = 2.9 × 10^−5^), while the FT4 PGS showed no association with thyroid cancer. Finally, a PGS specifically constructed for FT4 and atrial fibrillation including only 4 genetic variants (in *DIO1*, *LHX3*, *AADAT*, and *FOXE1*) (11) showed no association (OR = 0.84; *P* = .27), which could well be due to a limited number of included variants (48).

For a more accurate PGS for thyroid dysfunction, genetic variants from a dedicated GWAS on hypothyroidism and/or hyperthyroidism should be included. Including other disease risk factors, such as ethnicity, sex, age, and iodine status could further strengthen the prediction accuracy of PGSs (96, 97).

## Concluding Remarks and Future Perspectives

As genetic variation is the most important determinant of interindividual variation in thyroid function, it is key that the missing heritability gap is further narrowed. For this, the focus should shift toward rare variants, which can be studied in larger GWAS and in whole-exome and whole-genome sequencing studies, for which collaboration between large cohorts in international consortia (such as the ThyroidOmics Consortium) is quintessential. These studies should also include high-throughput in silico and in vitro analyses to identify novel genes and gene functions affecting TH regulation. Excitingly, recent studies have shown that genetics also has the potential to individualize the diagnosis and management of patients with thyroid diseases. Rather than investigating single genetic variants, future translational studies should focus on the combination of multiple genetic variants, environmental modifiers (eg, iodine status), and other nongenetic factors (eg, ethnicity, sex, age) in PGSs.

## Data Availability

Data sharing is not applicable to this article as no data sets were generated or analyzed during the current study.

## Abbreviations

BMI: body mass index
FT3: free triiodothyronine
FT4: free thyroxine
GWAS: genome-wide association studies
HPT: hypothalamic-pituitary-thyroid
LT4: levothyroxine
miRNA: microRNA
MR: mendelian randomization
OR: odds ratio
PGS: polygenic score
SNV: single-nucleotide variant
TH: thyroid hormone
TSH: thyrotropin
TT3: total triiodothyronine
